# Heavy Metals in the Environment and Thyroid Cancer

**DOI:** 10.3390/cancers13164052

**Published:** 2021-08-12

**Authors:** Fiorenza Gianì, Roberta Masto, Maria Antonietta Trovato, Pasqualino Malandrino, Marco Russo, Gabriella Pellegriti, Paolo Vigneri, Riccardo Vigneri

**Affiliations:** 1 Endocrinology, Garibaldi-Nesima Medical Center, Department of Clinical and Experimental Medicine, University of Catania, 95122 Catania, Italy; fiorenza.giani@gmail.com (F.G.); robertamasto88@gmail.com (R.M.); p.malandrino@unict.it (P.M.); mruss@hotmail.it (M.R.); g.pellegriti@unict.it (G.P.); 2 Surgical Oncology, Garibaldi-Nesima Medical Center, 95122 Catania, Italy; maritrov@icloud.com; 3 Medical Oncology and Center of Experimental Oncology and Hematology, Department of Clinical and Experimental Medicine, University of Catania, A.O.U. Policlinico Vittorio Emanuele, 95125 Catania, Italy; vigneri.p@unict.it; 4 Consiglio Nazionale delle Ricerche, Cristallography Institute, Catania Section, via P. Gaifami 18, 95126 Catania, Italy

**Keywords:** thyroid cancer, metal carcinogenesis, metals, metal pollution, metal mixture, thyroid stem cells, volcanic pollution

## Abstract

**Simple Summary:**

Epidemiological observations indicate that the incidence of thyroid cancer is increased in volcanic areas. Indeed, in the volcanic area of Sicily, where residents are biocontaminated by volcano-originated, low-level, multi-elemental metal pollution, the thyroid cancer incidence is double that in non-volcanic areas. The aim of this review is to summarize the evidence suggesting that chronic exposure to heavy metals, even at slightly increased environmental concentrations that cause no harm to mature thyrocytes, may alter the biology of stem/precursor thyroid cells, leading to a predisposition to malignant transformation. Both in vitro and in vivo experiments support this possibility; this phenomenon involves a variety of molecular mechanisms depending on the metal and the target cell involved. The role of the increased and generalized metal pollution in our ecosystem, paralleling the worldwide increase in thyroid cancer in recent decades, requires more attention and further studies.

**Abstract:**

In recent decades, the incidence of thyroid cancer has increased more than most other cancers, paralleling the generalized worldwide increase in metal pollution. This review provides an overview of the evidence supporting a possible causative link between the increase in heavy metals in the environment and thyroid cancer. The major novelty is that human thyroid stem/progenitor cells (thyrospheres) chronically exposed to different metals at slightly increased environmentally relevant concentrations show a biphasic increase in proliferation typical of hormesis. The molecular mechanisms include, for all metals investigated, the activation of the extracellular signal-regulated kinase (ERK1/2) pathway. A metal mixture, at the same concentration of individual metals, was more effective. Under the same conditions, mature thyrocytes were unaffected. Preliminary data with tungsten indicate that, after chronic exposure, additional abnormalities may occur and persist in thyrocytes derived from exposed thyrospheres, leading to a progeny population of transformation-prone thyroid cells. In a rat model predisposed to develop thyroid cancer, long-term exposure to low levels of metals accelerated and worsened histological signs of malignancy in the thyroid. These studies provide new insight on metal toxicity and carcinogenicity occurring in thyroid cells at a low stage of differentiation when chronically exposed to metal concentrations that are slightly increased, albeit still in the “normal” range.

## 1. Changes in Thyroid Cancer Epidemiology

In recent decades, the thyroid cancer incidence has increased more than most other cancers, including in both high- and low-income countries, and regardless of ethnic characteristics, environmental and lifestyle differences, and variable medical practices [1,2]. Because the majority of this increase concerns thyroid cancers of a small size and of the least aggressive papillary histotype, many experts believe that the continuous increase in thyroid cancer incidence is mainly due to the worldwide increased use of more sensitive diagnostic procedures, such as the ultrasound thyroid scan. The diffuse use of these diagnostic tools may have caused the emergence of a large reservoir of small, subclinical nodules that are histologically malignant but stationary or with a limited progression potential and that, therefore, will never or very rarely evolve into a clinical malignancy able to affect the patient’s health and survival [3]. Much indirect but strong evidence supports this possibility, and the issues of thyroid cancer “overdiagnosis” and “overtreatment” have been widely discussed in the last decade. The outcome has been new guidelines to avoid the potential harmful consequences and unjustified costs of excessive intervention [4,5].

There is evidence, however, that the worldwide increase in thyroid cancer incidence is not exclusively “apparent”, but that a “true” component is also contributing to the overall increase [6,7]. The observations that (a) thyroid-cancer-related mortality has not decreased despite earlier diagnosis and better treatment [8,9], and (b) the incidence of large thyroid tumors and anaplastic forms, which would have been discovered based on previous diagnostic techniques, has also increased [7,10,11] suggest that some environmental factors may have increased the risk of the initiation/progression of thyroid cancer. In recent decades, in fact, important changes in the environment and lifestyle have occurred, paralleling the increased diagnostic scrutiny. These changes may include underlying putative risk factors for thyroid cancer, a possibility supported by the changing pattern of thyroid cancer mutations over time [12,13].

A true increase in thyroid cancer incidence may originate from different factors. Exposure to one or more carcinogens specifically affecting thyroid follicular cells may have increased in recent decades, favoring malignant transformation with a prevalent effect on the papillary histotype. A well-recognized risk factor for thyroid cancer is the exposure to radiation, which, in fact, has increased in recent decades, mainly due to medical diagnostic procedures. Radiation could affect the thyroid more than other tissues because of high radio-sensitivity (especially at a young age) and also because of the frequent use of dental X-rays and head–neck–upper chest computed tomography (CT), which also irradiates the thyroid. Moreover, the widespread diagnostic use of iodine 131 (^131^I) in the 1970s and 1980s may also have played a role. Another thyroid-specific carcinogenic mechanism may derive from the iodine prophylaxis programs that have diffused throughout the world: iodine enrichment may increase autoimmune thyroiditis, which, in turn, may favor a thyroid cancer risk [14,15].

In recent decades, in addition to the aforementioned risk factors, a multitude of diet, lifestyle, and environmental pollutants may have affected the thyroid. Agroindustrial procedures, for example, have led to an increase in pesticides, repellents, and preservatives, whose chronic effect on the thyroid is unknown. Some of these elements, such as nitrites and nitrates ingested through drinking water contaminated by fertilizers and through processed meat consumption, have already been associated with thyroid cancer [16]. Other potential thyroid carcinogens include solvents, plastic constituents, and heavy metals derived from the increasing prevalence of the industrialized way of life. For most of them, no standards for safe values are available, and the effect on the thyroid of chronic, every-day exposure to these pollutants is difficult to evaluate. In any case, all evidence indicates that the causes of the true component of the worldwide increase in thyroid cancer are recent, environmental, and multiple [17]. The generalized low-level metal pollution of our environment meets all these requirements.

## 2. Volcanic Environment, Metals, and Thyroid Cancer

We recently described a marked increase in thyroid cancer in the volcanic area of Mt. Etna in Sicily [18], an observation that had already been reported in other volcanic areas [19,20,21,22]. In subsequent studies, we demonstrated that in the Sicilian volcanic area (a) the thyroid cancer incidence was doubled relative to adjacent non-volcanic areas in Sicily; (b) the thyroid cancer increase mainly concerned the papillary histotype, reflecting what has occurred regarding the worldwide increase in thyroid cancer incidence [18,23]; (c) there was a diffuse (water, atmospheric, and vegetal) environmental pollution with many metals [24,25]; and (d) residents of the area where the thyroid cancer incidence was increased were biocontaminated at a low level with numerous metals due to their lifelong exposure to the non-anthropogenically polluted volcanic environment [25,26]. We considered, therefore, that a better understanding of the possible carcinogenic effect of chronic human exposure to a multiple but low-level metal pollution in the environment and its effects on thyroid carcinogenesis could be obtained by studying this natural model of metal pollution.

Mt. Etna is a basaltic, continuously active volcano that hosts a major aquifer providing drinking and agricultural water to a large area with nearly 1 million inhabitants. In addition to the volcanic soil, the water from the volcanic aquifer is enriched with various metals due to the magmatic-type interaction of deep groundwater with gases and elements leaching from the volcanic rock [27]. Moreover, due to the continuous volcanic degassing, the atmosphere is polluted with a variety of metals, as documented by their bioaccumulation in lichens [25].

**Figure 1:**
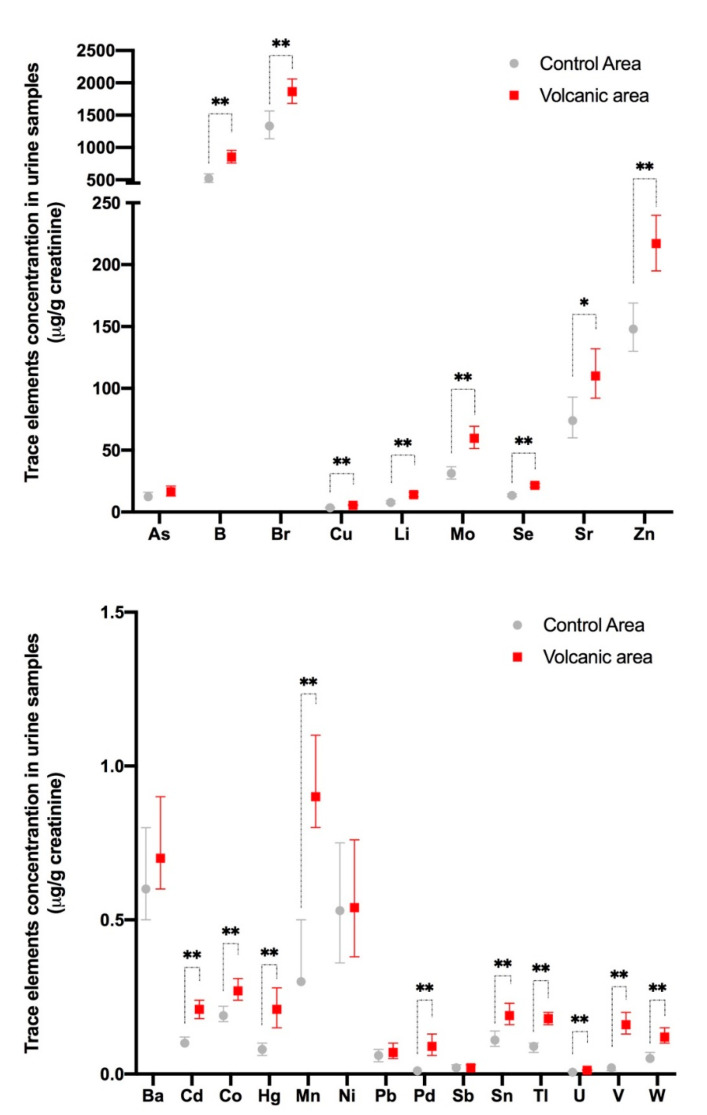
Metal concentrations in the urine of residents of the Mt. Etna volcanic and the control areas in Sicily. Twenty-three metals and metalloids were measured in the urine of 140 randomly selected permanent residents (F = 67.9%, mean age 48.1 ± 16.7 years) of the Mt. Etna volcanic area and 138 residents (F = 66.7%, mean age 46.3 ± 17.1 years) of adjacent non-volcanic control areas. Metal measurements were performed using an inductively coupled mass spectrometer and are expressed as µg/g creatinine. The values were not normally distributed and therefore the geometric mean was calculated for each specimen, and the values in each group were averaged to determine the mean value and its 95% confidence interval. There were statistically significant differences between the two groups based on linear regression analysis including the log-transformed values of each chemical. For all elements except As, Ba, Ni, Pb, and Sb, the values were significantly higher (* *p* < 0.05, ** *p* < 0.01) in the urine of volcanic area residents. Represented data are derived from ref. [25].

Residents of the volcanic area are chronically exposed to slightly increased levels of a cocktail of metals and, consequently, are biocontaminated. In fact, the concentration levels of many metals and metalloids (18 out of 23 examined) are significantly higher in the urine of these subjects than in the urines of residents in adjacent non-volcanic areas (Figure 1) [25].

There is variability in the urine concentration of single metals in different individuals, but only rarely are the values higher than cut-off values of the reference range. Indeed, only boron (B), molybdenum (Mo), palladium (Pd), and tungsten (W) urine values in the cohort of volcanic residents were higher than the 95th percentile of the reference values in more than 20% of the examined subjects. For most metals, the average increase in the urine of the residents of the volcanic area is small relative to values measured in the urine of residents of the control area: a twofold or more than twofold increase is present only for cadmium (Cd), mercury (Hg), manganese (Mn), Pd, thallium (Tl), uranium (U), vanadium (V), and W; however, in most cases the increase is low and for all metals examined the average value in the volcanic area is still within the normal limits [25].

As already mentioned, metal pollution in the Mt. Etna volcanic area is associated with an increased incidence of thyroid cancer [18,25,28], as has been described in other volcanic areas [19,20,21,22], although metals in the environment in those other areas have not been measured. The thyroid cancer increase in the Sicilian volcanic area is associated, although at a lower level, with the increase in other site-specific cancers [29,30], suggesting the possibility of a more generalized carcinogenic effect of the volcanic environment. This causal relationship between the volcanic environment and an increase in cancer is supported by more frequent DNA damage observed in residents of active volcanic areas [31]. On the basis of these observations, we hypothesized that there is a cause–effect relationship between the diffuse low-level metal pollution of the environment and the increased thyroid cancer incidence [32]. The present review aims to highlight the in vitro and in vivo evidence supporting the role of an increase in heavy metals in the environment as a likely cause contributing to the worldwide increase in thyroid cancer and to analyze the potential mechanisms involved.

## 3. Metal Interactions with Living Cells

Metals are naturally occurring elements that cannot be broken down, are not biodegradable, and are present in nature as trace elements. Many metals (copper [Cu], cobalt [Co], iron [Fe], selenium [Se], zinc [Zn], and others) at trace levels are necessary for life because they are essential for various metabolic processes and biologic signaling pathways. Other metals (arsenic [As], Cd, Pb, Hg, nickel [Ni], and others) are nonessential, do not have a biologic role in living cells, may be toxic even at very low concentrations, and are often classified as carcinogenic. However, when in excess, essential metals may also become toxic and carcinogenic.

Metal toxicity depends not only on the dose of the metal but as well on several physicochemical factors concerning each single metal and also from the biological characteristics of the target cells. Each metal, in fact, has unique chemical properties that determine its mechanism of action, while, on the other hand, each cell/tissue has specific biologic and functional characteristics that induce a particular response to metals. By interacting with toxic metals, cells may suffer two types of damage: direct damage caused by conformational and functional changes due to metals binding to molecules such as nucleic acids and enzymes, as well as indirect damage due to the metal-induced formation of reactive oxygen and nitrogen free radicals that in turn cause detrimental effects to the cell structures and biological functions. Cells activate two major protective mechanisms to defend themselves from metal toxicity: (a) metals can be bound and hidden within a protein or deposited in an insoluble form within intracellular granules; and (b) a variety of antioxidant systems can be activated to reduce the excess of metal-produced free radicals and rapidly restore a normal redox condition.

Metal pollution of the environment may occur because of natural causes such as volcanic activity, soil erosion, metal corrosion, and geological weathering. Today, however, metal pollution is mostly due to anthropogenic activities. In recent decades, human exposure to metals has risen dramatically as a result of the exponential increase in their use in several industrial, agricultural, technological, and domestic applications. A clear temporal trend of increasingly contaminated soil has been found for some metals [33,34,35,36]. Biocontamination with metals may occur by ingestion of contaminated food and water, by inhalation from the atmosphere, and by skin contact. Metal absorbance from the environment will depend on the chemical form, particle size, and solubility. Once a metal has entered the body, its toxicity will depend on binding with organic groups and cellular macromolecules and on increased reactive oxygen species (ROS)/reactive nitrogen species (RNS) production in different cells, affecting their chemical and functional characteristics.

Anthropogenic pollution and consequent human biocontamination can pertain to a single metal (often due to occupational reasons), but of greater and more general concern is the “silent” exposure of a population to the polluted industrialized environment and industrialized lifestyle. This increased exposure to metals is generalized (the entire industrialized world), chronic (lifelong), and involves multiple metals, usually at slightly increased levels, but because of the different combinations of the metal cocktails in different areas, the adverse consequences may be different. For these reasons, despite becoming a great concern for human health [37], the consequences of the generalized environmental pollution with metals are difficult to determine because the conditions are heterogeneous and our understanding of a problem that is threatening our ecosystem and our health is insufficient. Within this complex network determining metal toxicity and carcinogenic potential, very little information is available regarding the possible mechanisms involved in the effects exerted by slightly increased metal concentrations on thyroid cells.

## 4. Metals and the Thyroid

Metal toxicity can be organ/cell specific depending on the biological characteristics of cells involved. Follicular thyroid cells have a variety of specific peculiarities. First, because of their unique role for iodine uptake and thyroid hormone synthesis, thyrocytes are committed to live in the presence of a constant generation of hydrogen peroxide (H_2_O_2_) necessary for iodide oxidization. This process exposes cells to a permanently increased level of oxidative stress that is a risk factor for thyroid cancer [38]. Second, thyroid cells have a slow turnover time: human thyroid cells divide only five times during adulthood [39], and this long life may favor the accumulation of mutations due to exogenous insults. Third, in thyroid cells the mutation rate is 8–10 times higher than in other organs such as the liver or lungs [40], suggesting that thyroid cells may be more susceptible to the damage of exogenous toxicants. Metals could also preferentially damage the thyroid because of their increased bioaccumulation in the gland. The thyroid has specific mechanisms to uptake, store, and use iodine, but nothing is known about thyroid uptake of metals that, if accumulated, could reach locally toxic concentrations.

**Figure 2:**
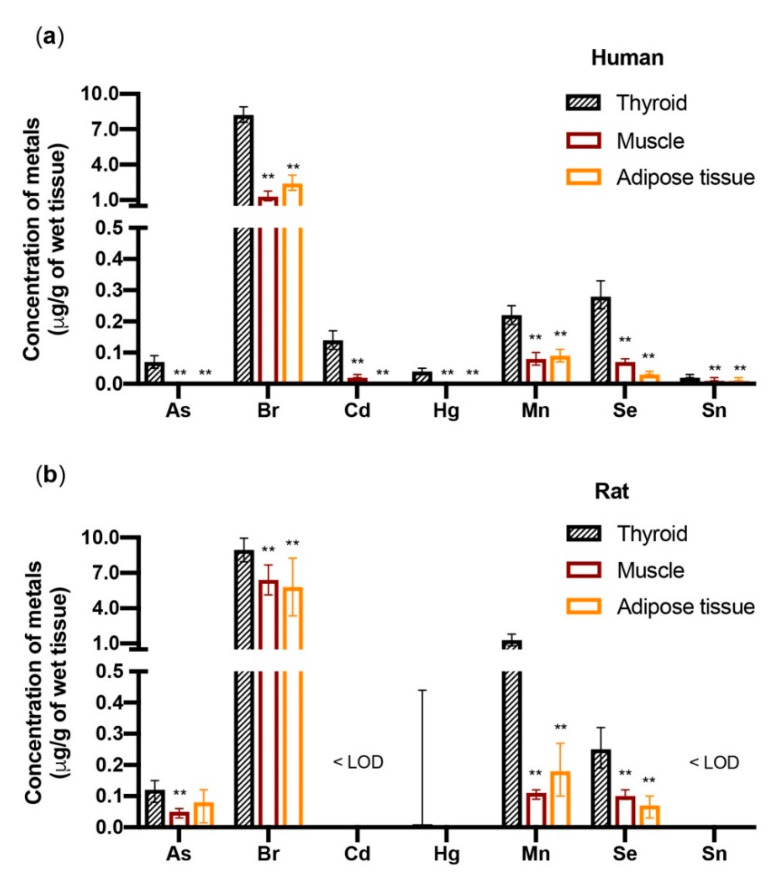
Metals accumulate in the thyroid more than in muscle and fat. The bars indicate the geometric mean and the 95% confidence interval of the metals (µg/g of wet tissue) in the tissues of (**a**) 77 euthyroid subjects (F = 70.1%) and (**b**) 8 female Wistar rats. Human specimens were collected from the thyroid, the sternothyroid muscle, and the neck subcutaneous fat. Rat specimens were collected from the thyroid, the hindlimb muscle, and the visceral abdominal fat. Only metals that accumulated in the human thyroid significantly (** *p* < 0.01) more than in muscle and fat are shown. In rats, but not in humans, Cu and Zn also significantly accumulated in the thyroid more than in the other two tissues (not shown, see ref. [41]). < LOD = below the limit of detection. Represented data are derived from ref. [41].

When the metal concentrations were measured in the thyroid as well as in muscle (sternothyroid muscle) and adipose tissue (subcutaneous neck fat) of the same euthyroid individuals, there was a significantly (*p* < 0.01) higher concentration of As, bromine (Br), Cd, Hg, Mn, Se, and tin (Sn) in the thyroid relative to the muscle and fat (Figure 2) [41].

In parallel experiments, there were higher concentrations of As, Br, Cu, Hg, Mn, Se, and Zn in the thyroid of Wistar rats relative to the muscle (hindlimb) and adipose tissue (abdomen visceral fat). The small differences observed between humans and rats may be due to species-specific biological differences but also to either the different muscle and fat tissue sampling or the different diet (free diet in humans vs. Mucedola 4RF21 in rats). Notably, three metals with recognized toxic and carcinogenic properties (As, Cd, and Hg) bioaccumulate in the human thyroid significantly more than in muscle and fat, raising the possibility that this increased accumulation can be relevant for the frequent occurrence of thyroid nodules due to either benign or malignant thyroid cell proliferation. These nodules are more frequent in the aged population, suggesting a possible role of a progressive metal accumulation with aging [42,43,44]. When the metal concentration in the thyroid of residents of the volcanic area (where the thyroid cancer incidence has doubled) was compared with values measured in residents of the control area, most metals (11/18) were higher in the former group; the differences, however, were not statistically significant [41], and no conclusion can be drawn regarding a specific role of metal concentration in the thyroid as a driving factor for the increased thyroid cancer incidence in the volcanic area.

## 5. Effects of Metals in Mature and Immature Thyroid Cells

### 5.1. In Vitro Studies with Single Metals

A major challenge in assessing a cause–effect relationship between human exposure to environmental metal pollution and cancer is the non-standardized environmental conditions and the long follow-up required to collect precise biochemical and biological measurements in a large number of exposed subjects. In vitro experiments in cultured cells and in vivo studies in experimental animals are practicable alternatives to explore this problem. The increased incidence of thyroid cancer in the Mt. Etna metal-polluted area is associated with the residents’ exposure to increased metals that is (a) lifelong, (b) low-level, and (c) multi-elemental. Taking in account these characteristics, we investigated the potential cause–effect relationship between metal exposure and thyroid effects both in vitro and in vivo.

In vitro, there were no effects in human thyroid cell morphology, viability, or proliferation when thyrocytes in primary cultures were chronically (72 h) exposed to several heavy metals (Cd, Cu, Hg, Mn, Pd, W, and Zn) at concentrations that are 0.1–100-fold relative to those measured in the urines of residents of the volcanic area. Based on previous observations [45,46,47,48] that immature progenitor cells may be more sensitive to the toxic effect of pollutants, we tested the same heavy metals, at the same low concentrations, in undifferentiated human thyrocytes in the form of thyrospheres. Thyrospheres are aggregates of thyroid stem cells and precursors of thyrocytes at a different level of differentiation [49] that, depending on the culture conditions, are able to differentiate into mature thyrocytes or produce additional thyrospheres by a process involving stem cells and named self-renewal [50]. Chronic exposure of thyrosheres to different metals (Cu, Hg, Pd, W, and Zn) at a very low dose significantly increased proliferation (BrdU incorporation) with a biphasic dose-response curve: in all cases the values progressively increased, reached a peak (+35% to +59% relative to the control thyrospheres for the different metals), and then declined when the metal concentration was further increased (Table 1) [51].

This low-dose bimodal response is typical of hormesis [52], a biological phenomenon occurring at very low concentrations of a substance and already documented for many chemicals, including metals [53,54], both in vitro and in vivo.

For most metals, the hormetic effect is observed in the micromolar concentration range [45,55]. However, the biological response depends not only on the concentration of the metal studied but also on the time of exposure and the type of target cell/tissue examined. No previous data on the hormetic phenomenon are available for mature or immature thyroid cells. The hormetic response to chronic metal exposure in human thyrospheres occurred in the nanomolar concentration range; the same level measured in the urine of residents of Mt. Etna volcanic area (see Figure 1).

**Figure 3:**
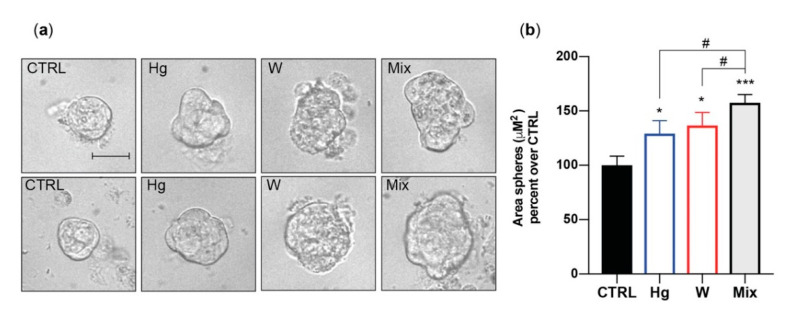
Morphological changes induced by thyrosphere exposure to metals. (**a**) Representative phase-contrast images of thyrospheres grown for 8 days in standard medium (CTRL) or in medium with either 0.1 nM of HgCl_2_ or 30 nM Na_2_WO_4_. The metal mixture (MIX) included the metal salts of Cu, Hg, Pd, W, and Zn at the concentration causing the greatest effect on BrdU incorporation (see Table 1). The scale bar is 30 µM. (**b**) Average values of the thyrosphere size calculated by measuring sphere areas using the Image J software from NIH. The bars indicate the mean ± standard error of the mean of three independent experiments (* *p* < 0.05, *** *p* < 0.001 (relative to CTRL), # *p* < 0.05 (Hg and W vs. Mix)). Represented data from ref. [56].

In vitro, therefore, all examined heavy metals, at environmentally relevant concentrations, caused thyroid stem/precursor cell growth but had no effect on mature thyrocytes. Phase-contrast imaging indicated that the metal-induced growth effect concerned the size rather than the number of exposed thyrospheres, suggesting that metals stimulated the proliferation of partially differentiated thyroid cell precursors rather than self-renewal of stem cells. In addition, imaging analysis showed that metal-exposed thyrospheres were not only larger but also showed an irregular morphology, suggesting that the presence of increased metals also affected the orderly thyroid stem/precursor cell aggregation into spheres (Figure 3) [56].

The biological consequences of such abnormalities are unknown. Overall, stimulation of immature thyroid cell growth is a shared effect of all the heavy metals tested, but this does not mean that the mechanism is similar for all metals or that this is the only biological effect.

To carry out a wider investigation of the biological consequences of chronic exposure to slightly increased metal concentrations, the effects of W, the metal with the most increased concentration in the water of the Mt. Etna volcanic area relative to the water of adjacent non-volcanic areas, was investigated. W is an emerging toxicant with potential carcinogenic effects both in vitro [57,58] and in vivo [59]. Its use has greatly increased in the industrialized world in recent decades with consequent greater contamination of the environment [60]. Its effects on human thyroid cells have never been investigated.

In vitro chronic exposure of human thyrospheres to nanomolar concentrations of soluble W in the chemical form of sodium tungstate dehydrate (Na_2_WO_4_2H_2_O) not only stimulated proliferation (as already shown in Table 1), but had the following effects [61]:

It inhibited apoptosis, as indicated by a blunted caspase 3/7 increase after staurosporine and raptinal application.

It changed the expression of several genes, with an increase in some genes that are abnormally expressed in thyroid cancer such as ATP-binding cassette subfamily G member 2 (*ABCG2*) [62], c-Myc (*MYC*) [63], and forkhead box protein A2 (*FOXA2*) [64].

It increased the phosphorylation of the DNA-repair histone γH2AX and increased the nuclear translocation of the protein 53BP1; both proteins are markers of double-strand DNA breaks and DNA damage [65,66].

It reduced stem/precursor thyroid cell differentiation, as indicated by a significant increase in the stemness genes octamer-binding transcription factor 4 (*OCT4*) and *ABCG2* and a decrease in the thyroid-specific differentiation genes thyroglobulin (*TG*), thyroid peroxidase (*TPO*), and thyroid-stimulating hormone receptor (*TSHR*). These changes were present not only in W-exposed thyrospheres, but also in the derived mature thyrocytes [61].

It influenced the biological characteristics of mature thyrocytes derived from W-exposed thyrosheres: these secondary thyrocytes showed signs of cell transformation such as forming more and larger colonies in soft agar and in the clonogenic assay and exhibiting a greater migration capacity, all signs of cell transformation.

These data indicate that chronic exposure to a low dose of soluble W not only affects thyroid stem/precursor cell biology, but it may also cause abnormalities in their differentiated progeny. Increased proliferation, decreased apoptosis and differentiation, and abnormalities in DNA-repair proteins are changes compatible with a transformed state. These studies suggest, therefore, that mature thyrocytes that originated from chronically W-exposed progenitors may represent a thyroid cell population susceptible to neoplastic transformation. When translated to the clinic, the lifelong exposure to slightly increased toxic metals, occurring also during the prenatal and the early life period, may be an underestimated matter of concern for the increased incidence of thyroid cancer in residents of a volcanic area.

### 5.2. In Vitro Effects of a Metal Mixture

The in vitro cell model used to study the effect of single metals on thyroid cells does not necessarily reflect what will occur in vivo. In addition to the well-known in vitro versus in vivo differences, an additional major difference concerns the fact that in the real world, metals are generally present as multiple components of the environmental pollution. Individuals living in a metal-polluted environment, such as the Mt. Etna volcanic area, are exposed to a mixture of different metals, most at a slightly increased concentration. To address this problem, we used a simplified in vitro model by chronically treating thyrosheres with a mixture of Cu, Hg, Pd, W, and Zn, each at the concentration eliciting the maximum response in thyrosphere growth stimulation (see Table 1). Chronic exposure to the mixture of Cu, Hg, Pd, W, and Zn caused a significantly greater effect on BrdU incorporation (91.0% ± 14.8%) than caused by each metal acting alone [56]. Moreover, while single metals had an effect only on thyrosphere size, their mixture increased also the thyrosphere number, suggesting not only a more potent growth stimulation, but also a different mechanism of action. No effect of this metal mixture was observed in mature thyrocytes.

The effect of multiple metals, acting simultaneously on a biological target, depends on many factors, including the relative abundance of each metal and the possible additive (i.e., ROS/RNS production), antagonistic (i.e., competition for binding to organic components or for membrane transporters), or synergistic (i.e., activation of different enzymes along the same signaling pathway) interactions between the different effects of the components of the metal mixture. Considering that the number of possible combinations of metals is potentially enormous (both in terms of the metals involved and their concentration), studying the combined effect of metals is a daunting challenge, and it is not possible to test every possible mixture.

Mathematical methodologies have been developed to estimate the consequences of the combined effect of chemicals: These conceptual models are mainly based on the assumption of either additive or independent actions, and they have been used to predict the mixture toxicity and to obtain some insight on the mechanisms associated with metal mixture [67,68,69]. These mathematical models, however, have limited practical applications because of the assumption that there is no interaction among the components of the mixture and because it is difficult to consider the relevance of the different chemical speciation of each metal present in the mixture [70].

Our experiments with immature thyroid cells chronically exposed to the mixture of five metals confirm, in a simplified model, the complexity of assessing the effects of a metal mixture and highlight that the increased biologic and potentially toxic effect of a metal mixture occurs also when metals are present in the mixture at only slightly increased concentrations.

## 6. In Vivo Studies in Experimental Animals

Animal and human exposure to excess concentrations of metals is known to cause toxic and carcinogenic effects. These detrimental actions of metals have been demonstrated on many endocrine glands, including the thyroid [71,72,73]. Although chronic exposure to increased heavy metals in the environment favors the risk of developing various types of cancer [74,75], most studies regarding the thyroid have rather focused on the effect of metals on thyroid function or thyroid hormone action [76,77]. Moreover, metal concentrations in malignant versus normal thyroid tissue have also been investigated as possible markers of malignancy [78,79,80,81]. By contrast, in vivo thyroid carcinogenesis due to exposure to excess metals in the environment has been poorly studied and never as the possible consequence of exposure to only slightly increased metal concentrations.

To investigate the above-mentioned issue, we used a well-established model for studying in vivo thyroid carcinogenesis. Female Wistar rats were made prone to develop goiter and thyroid cancer because of severe hypothyroidism induced with chronic treatment with methimazole and a low-iodine diet [82,83]. The experimental rats were then divided into two groups: a control group and a metal-treated group. In the latter, three metals, B, Mo, and Cd, were added to the drinking water at a concentration double that measured in the urine of the residents of the Mt. Etna volcanic area [84]. These metals were chosen not only because they were markedly elevated in the water of the volcanic area relative to the water of the control area in Sicily (Mo and Cd), but also because they were among the few metals exceeding the urine reference limit in more than 20% of residents of the volcanic area (B) [25]. All three metals have already been reported to influence, at higher concentrations and in different models, thyroid biology [76,85,86,87].

At the low dose used, the three-metal mixture caused no overt toxic effect in animals as judged by food and water consumption and by animal weight increase. Metal intake was documented by the significantly increased concentration in the urine of the treated animals, in which the metal concentration reached the same range of values that had been added to the drinking water, suggesting no major retention/accumulation effect. As expected, due to the low-iodine diet, urinary iodine was greatly and similarly decreased in the rats of both groups.

After 5 and 10 months, the rats were sacrificed to measure the metal concentrations and to evaluate any histopathological changes in the thyroid. As a consequence of the induced hypothyroidism, the thyroid was markedly enlarged in all rats without a difference between the two groups, and showed histological abnormalities typical of hypothyroidism (irregular follicular pattern with microfollicles and cuboidal cells). At 5 months, there were only small differences between the two groups, with only slightly increased inhomogeneity in the treated animals. After 10 months, however, there were marked differences in the histological morphology of the thyroid: In addition to an evident disorganization of the follicle structure and the presence of hyperplastic and oxyphilic cells (both signs typical of chronic hypothyroidism caused by goitrogens), there were more papillary structures in the thyroid (present in all metal-treated animals vs. only one case in the control group). In addition, abnormal nuclear morphology and nuclear pseudo-inclusions were present in the thyroid of metal-treated animals [84]. B and Mo concentrations in the thyroid were not different between the two groups, while Cd was undetectable and the iodine content was reduced in the thyroid of treated animals, suggesting a possible chronic influence of the metal mixture on the thyroid iodine uptake or retention [84].

These studies indicate that, in an in vivo model prone to develop thyroid cancer, the long-term exposure to a mixture of B, Cd, and Mo in the non-toxic range increased the appearance of histological abnormalities that are markers of follicular cell malignant transformation. These deleterious effects due to low-level metal concentrations require a long-lasting exposure (many months) to become evident. It is not clear whether the carcinogenic effect was due to only one of the three metals studied or to the metal mixture, and the possibility of an indirect carcinogenic effect secondary to the reduced iodine content in the thyroid cannot be excluded.

## 7. Molecular Mechanisms of Action

The molecular mechanisms of metal effects have been widely studied with very different results depending on the individual metal characteristics, the dose used, the time of exposure, and the biological model studied. Metals may have a multiplicity of biological effects on cellular signaling pathways. In addition to the metal-induced oxidative stress, metals can directly influence a cell’s biology by a variety of mechanisms, including: (a) binding and activating cell surface transporters and receptors [88,89]; (b) modulating selected intracellular kinases and phosphatases [90,91]; (c) activating metallothioneins [92,93] and specific enzymes [94]; and (d) inducing DNA damage or affecting DNA repair systems [95]. Within this complex network determining metal toxicity and carcinogenicity, very little information is available on the mechanisms involved in the effects of metals on thyroid cells when they are present at only slightly increased concentrations.

**Figure 4:**
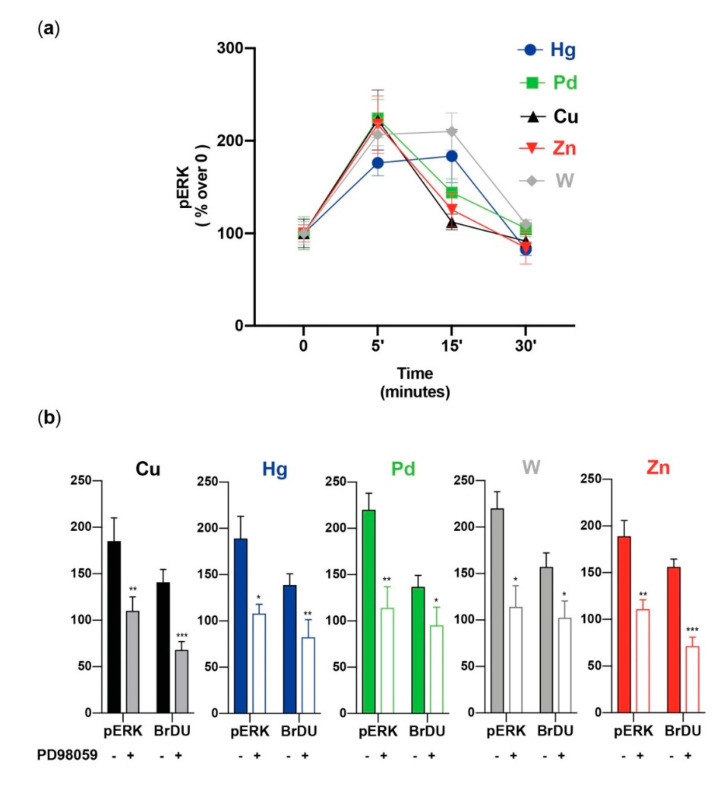
Metal-induced ERK1/2 phosphorylation and proliferation. (**a**) Time course of ERK1/2 phosphorylation in thyrospheres exposed to metals at the concentration causing the greatest BrdU incorporation (see Table 1). The mean ± standard error of the mean of six separate experiments are shown. In all cases, the metal-induced increase was significant at 5 min, when it reached the peak value (except for Hg and W, which remained elevated for up to 15 min). (**b**) The effect of the ERK1/2 inhibitor PD98059 on ERK1/2 phosphorylation and on thyrosphere proliferation (BrdU incorporation) after cell exposure to the indicated metals. In all cases, the significant inhibition of ERK1/2 activation was associated with a significant inhibition of BrdU incorporation. The bars indicate the mean ± standard error of the mean of three separate experiments (* *p* < 0.05, ** *p* < 0.01, and *** *p* < 0.001). Represented data from ref. [56].

Extracellular signal-regulated protein kinase (ERK1/2) and protein kinase B (PKB or AKT) are major components of intracellular signaling. Both are especially important in regulating cell proliferation [96,97,98,99], and both are sensitive to the presence of increased/decreased metal concentrations [98,99,100,101,102]. Therefore, we evaluated whether the metal-induced proliferation of stem/precursor thyroid cells involves the activation of these pathways. All five metals tested (Cu, Hg, Pd, W, and Zn), at the same environmentally relevant concentrations causing thyrosphere proliferation, rapidly (peak value at 5–15 min) and markedly (*p* < 0.01 for all) increased ERK1/2 phosphorylation (Figure 4).

As already observed for proliferation, the metal mixture caused a significantly greater increase in ERK1/2 phosphorylation than any of the metals alone. Under the same conditions, the metals had a minimal, non-significant effect on AKT phosphorylation. Again, this was not the case for the metal mixture: there was a significant AKT phosphorylation elicited in thyrospheres exposed to the five-metal combination. The same metal concentrations used for thyrosheres did not stimulate ERK1/2 and AKT phosphorylation or cell growth in mature human thyrocytes. The use of the ERK1/2 inhibitor PD98059 confirmed the major role of ERK1/2 activation on the metal-induced thyrosphere growth. Pre-incubation of thyrospheres with this inhibitor not only blocked ERK1/2 phosphorylation, they also reduced thyrosphere proliferation (Figure 4). These effects occurred for each of the five metals tested, although with a variable magnitude, with a greater effect for Cu, Zn, and the metal mixture [56].

Our observations indicate that, in stem/precursor thyroid cells, the ERK1/2 signaling pathway plays a major role in the growth caused by the hormetic metal stimulation. It has already been reported that most cell types utilize the ERK1/2 signaling pathway to activate the hormetic dose-response to promote proliferation [103]. However, the correlation between ERK1/2 activation and thyrosphere proliferation that we observed for all metals does not imply an identical mechanism of action. The upstream steps leading to the common activation of the ERK1/2 pathway may be very different for distinct metals and cells because different mediators can be activated by different metals and then stimulate the intracellular pathways. Zn, for example, can directly activate a specific surface G-protein coupled receptor (GPCR) and then the ERK1/2 pathway [88]. W can elicit a similar GPCR-mediated mechanism in cultured hepatocytes [104] and in thyrospheres [61]: in the presence of pertussis toxin (a GPCR inhibitor), W had a reduced effect on both ERK1/2 activation and thyrosphere proliferation.

The metal-activated mediators leading to ERK1/2 phosphorylation can be cell-specific and dose-dependent: at 1 mM, W activated the BKαβ1 channels and ERK1/2 phosphorylation in HEK cells [105], but at 30 nM it had no effect on BKαβ1-mediated ERK1/2 activation in human thyrospheres [61]. Moreover, the metal dose may have bidirectional effects on ERK1/2 signaling, as documented for various metals [106,107]. Similar bidirectional effects may also depend on short- or long-term exposure to toxic metals [107], indicating that the final biological effect depends on the magnitude and duration of the exposure: the cell–metal interaction will cause either the predominance of detoxification and cell adaptive processes allowing (deregulated) survival or, when cell defenses are overwhelmed, may lead to cell death.

## 8. Conclusions

The progressive worldwide increase in the incidence of thyroid cancer that has been observed in recent decades parallels the increase in human exposure to heavy metals in the environment, a consequence of the industrialized way of life. Chronic exposure to an even modest increase in metal pollution may lead to a variety of toxic effects in different tissues. By studying a natural model of slightly increased environmental pollution with multiple metals—the Mt. Etna volcanic area—we observed the association between metal pollution, residents’ biocontamination, and an increased incidence of thyroid cancer. These observations are compatible with a cause–effect relationship between metal pollution and thyroid cancer. The hypothesis that toxic metals may influence the malignant transformation of thyroid cells had already been proposed many years ago [108]. The now-available in vitro and in vivo studies support this possibility and suggest that thyroid stem/precursor cells rather than differentiated thyrocytes are the major target for the toxic effects of low-level metal pollution. This is the most relevant and novel finding: Low-level increased metal concentrations that cause no harm in mature thyroid cells significantly alter the biology of thyroid stem/precursor cells, a likely consequence of the different genetic characteristics and different capacity to respond to the oxidative stress injury of cells at different stages of differentiation. This observation could translate into clinical consequences: during sensitive life stages (such as prenatal life and early childhood), metals in the environment could cause thyroid stem cell damage at a low level of exposure that would have little or no adverse effects in well-differentiated thyroid cells. As suggested by previous studies in hormone-sensitive tissues like the prostate [46] and the thyroid [109], stem/progenitor cells may be the long-lived targets of toxicants that alter their differentiation capacity and increase their transformation susceptibility. Preliminary in vitro evidence with W indicates that thyroid cell progenitors exposed to slightly increased metal concentrations may be the origin of mature thyrocytes with abnormal characteristics typical of transformed cells [61]. Our studies also indicate that (a) the metal effects on stem/precursor cells, occurring at slightly increased levels, still in the range of environmental reference values, require a long exposure (days or weeks in vitro and many months in vivo), and (b) the thyroid may be specifically affected because of its biological characteristics and because some carcinogenic metals may accumulate in the thyroid more than in other tissues such as muscle and fat.

Another relevant finding is the increased effect of a metal mixture compared with each individual component at the same concentration. This observation could also be clinically relevant because the generalized condition present in real life is the population exposure to a mixture of increased metal concentrations, a condition that has progressively worsened in recent decades, paralleling the increasing incidence of thyroid cancer.

The molecular mechanisms of action remain largely undetermined but are certainly heterogeneous, because many factors are involved and the biological effects are different depending on the metal/mixture examined and the many possible specific conditions [110]. We documented the activation of a common pathway for promoting immature thyroid cell proliferation and deregulated biology following chronic exposure to low-level increased metals. The stimulation of ERK1/2 phosphorylation, however, is probably a shared segment of a complex network including a multiplicity of upstream and downstream components.

In conclusion, we believe that the present evidence provides biological plausibility to the hypothesis that environmental metal pollution, even at a low level, plays a role in the propagation of altered thyroid stem/progenitor cells, leading to a progeny of transformation-prone mature thyrocytes that could favor an increased risk of thyroid cancer on the basis of future carcinogenic events and/or in genetically predisposed individuals [111,112,113]. This hypothesis requires long-term in vivo studies of metal toxicity and carcinogenesis [114] on the thyroid using a metal mixture with individual metal doses at environmentally relevant concentrations (lower than the present standard of maximum tolerated concentration). Moreover, the experimental model should include animals generated in a metal-exposed environment to better mimic what occurs in our metal-polluted ecosystem.

We hope that these novel findings will inspire future epidemiological, etiological and clinical studies aimed at further clarifying the relationship between environmental metal pollution and thyroid cancer.

## Figures and Tables

**Table 1:** Chronic exposure (3 days) to the indicated metals increased proliferation (BrdU incorporation) in human thyrospheres (stem/precursor thyroid cells) but not in mature thyrocytes in primary culture.

**Cu** (16 nM)	CuSO_4_ (nM)	1	10	100	1000
	Thyrospheres	120.3 ± 10.3	136.7 ± 6.9 ***	128.2 ± 7.8 **	111.3 ± 6.1
	Thyrocytes	101.6 ± 8.3	113.9 ± 15.0	103.2 ± 7.1	98.7 ± 7.5
**Hg** (0.02 nM)	HgCl_2_ (nM)	0.001	0.01	0.1	1
	Thyrospheres	108.0 ± 6.7	122.4 ± 7.0 *	137.0 ± 10.16 **	84,94 ± 9.6
	Thyrocytes	102.5 ± 11.44	101.7 ± 7.0	104.3 ± 5.1	98.3 ± 12.45
**Pd** (0.03 nM)	PdCl_2_ (nM)	0.001	0.01	0.1	1
	Thyrospheres	129.7 ± 6.7 **	136.2 ± 7.2 ***	120.9 ± 7.4 *	114.5 ± 7.3
	Thyrocytes	92.8 ± 14.5	90.7 ± 6.0	104.1 ± 8.7	74.8 ± 8
**W** (1.1 nM)	Na_2_WO_4_ (nM)	1	10	100	1000
	Thyrospheres	129.9 ± 7.5 *	159.5 ± 13.0 **	158.5 ± 18.6 *	109.7 ± 8.8
	Thyrocytes	106.4 ± 8.6	102.5 ± 7.4	97.2 ± 9	99.7 ± 11.3
**Zn** (47 nM)	ZnCl_2_ (nM)	10	100	1000	10,000
	Thyrospheres	130.8 ± 10.8 *	158.8 ± 10.1 ***	162.2 ± 13.3 **	133.4 ± 11.3
	Thyrocytes	100.8 ± 10.5	116.5 ± 10.13	98.8 ± 13.32	97.1 ± 11.26

The data indicate the mean ± standard error of the mean percent change relative to the basal level of unexposed cells (always equal 100) considering four separate experiments. The average metal concentration in the drinking water of the volcanic area in Sicily is also indicated (in parentheses). All metals, used at a very low concentration, caused a biphasic response typical of hormesis. The values in bold indicate the peak value for each metal. * *p* < 0.05, ** *p* < 0.01 and *** *p* < 0.001 relative to the basal value. Represented data are derived from ref. [56].

## Data Availability

Not applicable.
